# Supplementary material for: Rapid earthquake magnitude classification via P-wave strains from borehole strainmeters and Distributed Acoustic Sensing
Source: Nat Commun. 2026 Jun 3;17:4776. doi: 10.1038/s41467-026-72223-z (PMC13233862; doi:10.1038/s41467-026-72223-z)
Supplement: Supplementary file 1 — Supplementary Information [file 41467_2026_72223_MOESM1_ESM.pdf]

Supplementary Information

Rapid earthquake magnitude classification via P-wave strains from borehole strainmeters and Distributed Acoustic Sensing

Sawi, T. M.[1], McGuire, J. J.[2], Barbour, A. J.[3], Yoon, C. E.[4], Karrenbach, M.[5], Stewart, C.[6]

1. U.S. Geological Survey, Earthquake Science Center, Moffett Field, CA, USA, tsawi@usgs.gov
2. U.S. Geological Survey, Earthquake Science Center, Moffett Field, CA, USA, jmcguire@usgs.gov
3. U.S. Geological Survey, Earthquake Science Center, Vancouver, WA, USA, abarbour@usgs.gov
4. U.S. Geological Survey, Earthquake Science Center, Pasadena, CA, USA, cyoon@usgs.gov
5. Seismics Unusual, LLC., Fullerton, California, USA, martin.karrenbach@seismicsunusual.com
6. Office of the President, California State Polytechnic University, Humboldt, Arcata, CA, USA, connie.stewart@humboldt.edu

Borehole Strainmeter Data Processing Steps

Raw strain data are downloaded from EarthScope Consortium and converted to linear strain by the vector operations:

$$\left(\frac{100e-6}{87e3}\right) * \left(\frac{\boldsymbol{t}}{1e8}\right) / \left(1 - \left(\frac{\boldsymbol{t}}{1e8}\right)\right),$$

where $\vec{t}$ is the raw waveform vector data (56). Missing data are replaced with nans and the remaining data are de-meaned by subtracting the mean from the vector data.

Supplementary Data 1. Naming scheme (short name and description) for all 127 features used in first iterations of X The numbers after “Morlet” are the center frequency and the bandwidth frequency, where the center frequency determines the primary frequency the wavelet is designed to detect, and the bandwidth frequency determines how wide the wavelet is, with a larger number indicating a wider, less focused wavelet.

Feature	Weighted_Count
pow1_max_1	35.274
pow1_max_0	16.497
pow1_max_9	11.815
pow2_range_0	10.556
pow2_range_4	10.187
maxAmp_avg_max	10.175
pow1_range_0	6.908
pow1_max_2	5.197
pow3_max_9	3.842
pow1_max_6	3.058

Table S1. Top ten features based on SHAP feature importance metric from each of the 50 model runs of first round of XGBoost. "Weighted Count" is the inverse rank weights. Naming scheme for features is explained in Table S1 (above).

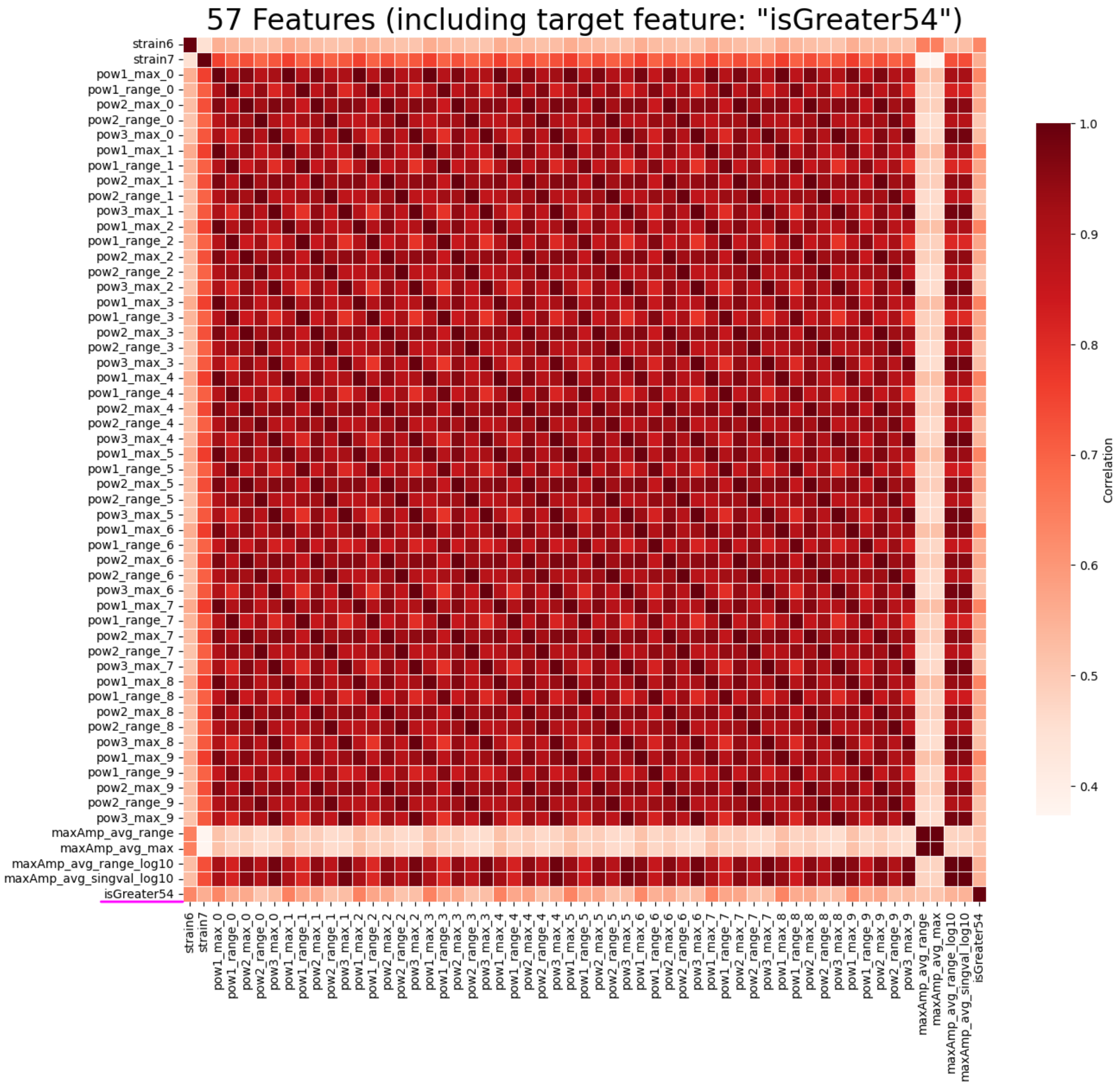

Figure S1. Correlogram showing 56 features whose absolute correlation with target feature (57th feature, underlined in fuchsia on vertical axis), calculated from all strainmeter waveforms, is more than 0.5. Correlations are calculated as $r_{xy} = \frac{Cov(X,Y)}{\sigma_X \sigma_Y}$, where r_{xy} is the correlation coefficient, $Cov(X,Y)$ is the covariance between the two features, and σ is the standard deviation. “Max” or “range” indicates whether the scalar feature is the maximum value or the range of the time series of the mean power of the coefficient of the continuous wavelet transform in the frequency band (if the feature begins with “pow”) or of the strain waveform across the 10 sliding windows (see “Methods”).

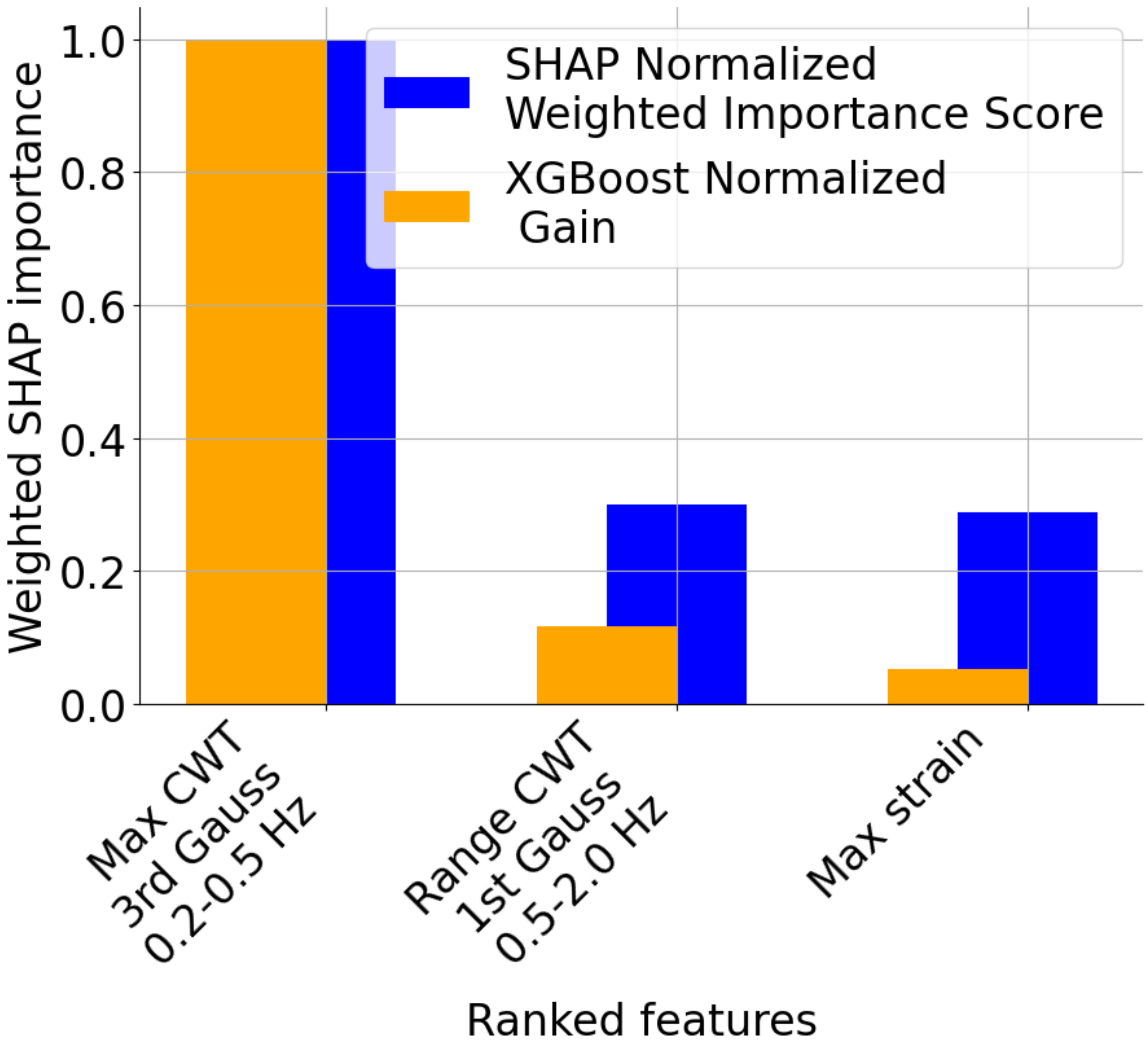

Figure S2. Comparison of SHAP feature importance score (blue) and XGBoost gain (orange), both normalized from zero to one. XGBoost gain describes how well each feature improved a given split, averaged over all splits in the model. SHAP scores, on the other hand, describe how a given feature impacts the prediction overall.

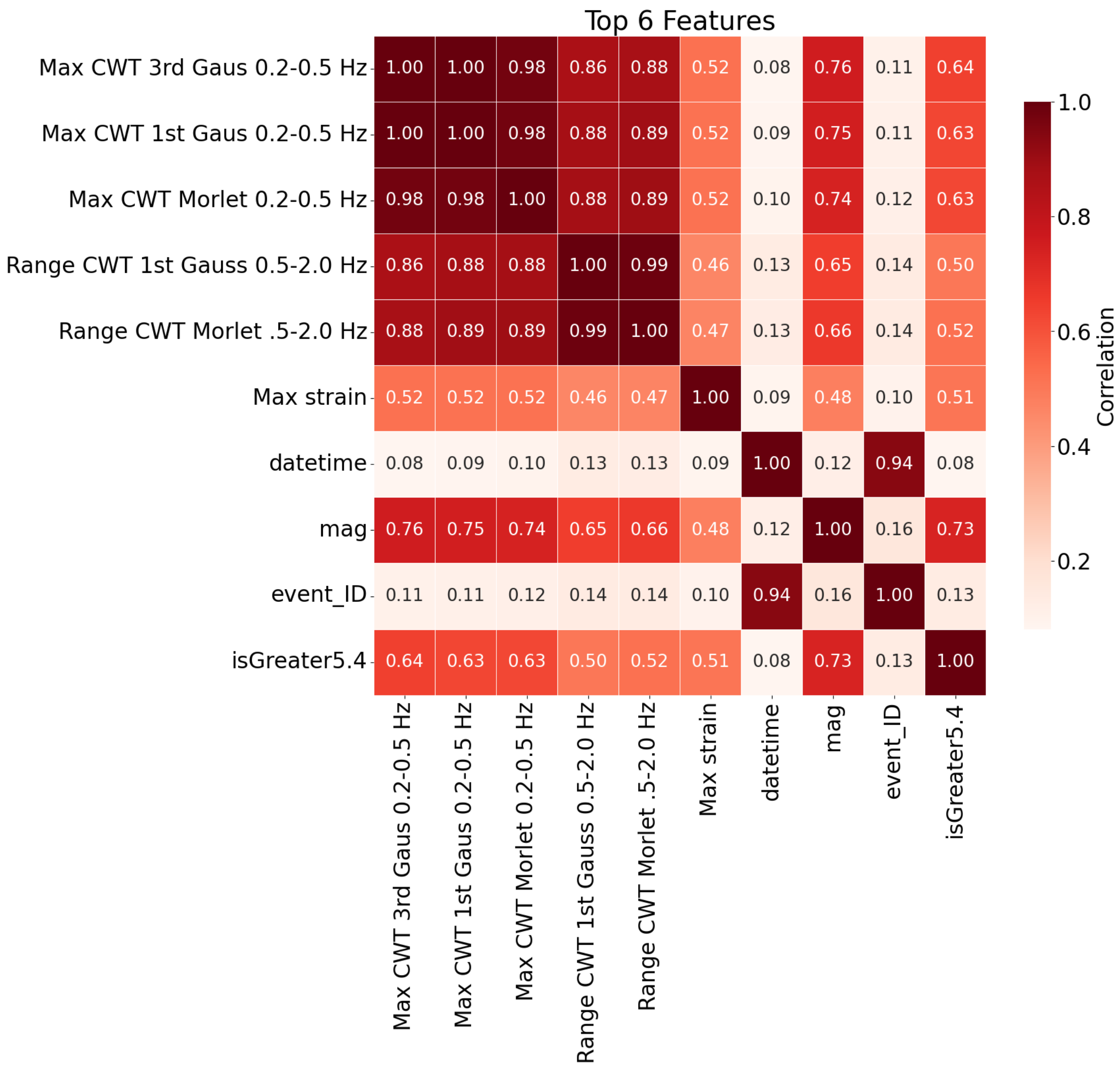

Figure S3. Same as Figure S1, but with only the 6 features with the highest SHAP feature importance metric.

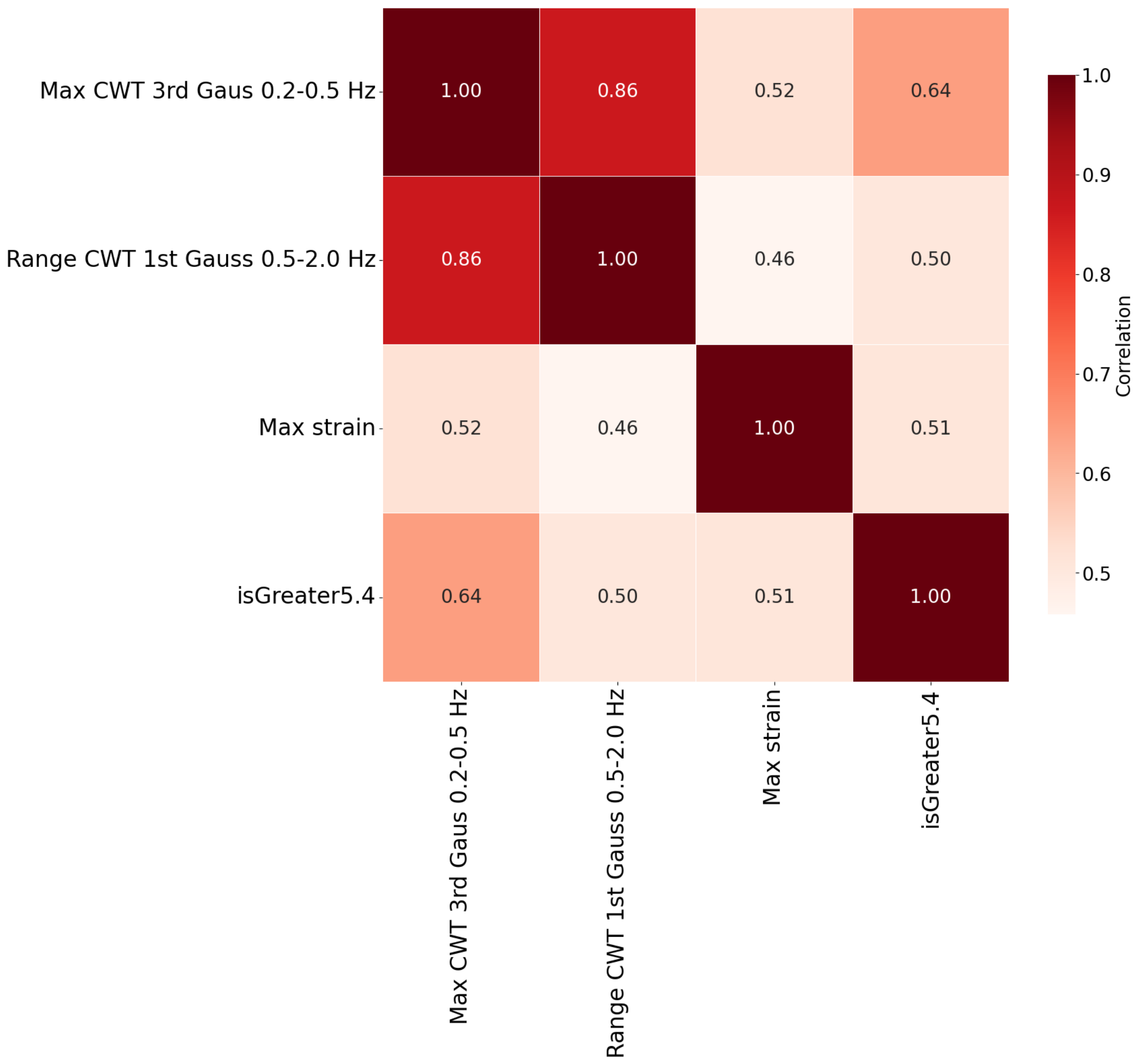

Figure S4. Same as Figure S1, but with the 3 features that are less correlated with each other.

Description of a decision tree:

At the top of the tree (Figure 4) is an example of a node, where data is split based on a Boolean condition. One feature ("pow1_max_1") is chosen randomly from a subset of features, as is a Boolean condition (" <=-129.373"). The test set is divided into two groups, or, "values", based on this Boolean. In this example, 656 of the 682 members of the test set are True with respect to the Boolean, and 26 are False, so the node is classified as the positive (True) class, "is >= M5.4." A sequence of descending nodes is called a branch. A subset of each of the True and False values are sent to the left and right branch, respectively, where the process is repeated until a terminal node ("leaf") is reached, where the final classification for those values is decided. A "gini" score is calculated for each node as $1-\Sigma(p_i)^2$ where p_i is the probability of each data point blowing to class "i" within a given node, so that in the top node in example in (a), the gini score = $1 - ((656/682)^2 + (26/682)^2) = 0.073$. A lower gini score indicates a better, more distinct classification. A leaf node is determined either by the user choosing the depth of the tree (that is, how many iterations of nodes to use, in this example, three) or until the gini score reaches 0.

Table S2. First round XGBoost grid search parameters.

Parameter	Values
learning_rate	0.001, 0.01, 0.1
n_estimators (number of trees)	1000, 2000, 4000
max_depth	3, 6, 9
scale_pos_weight	len(y_train[y_train == 0]) / len(y_train[y_train == 1])
colsample_bytree (i.e., fraction of features used by tree)	1

Table S3. Second round XGBoost parameters for final predictive model

Parameter	Values
learning_rate	0.1
n_estimators (i.e., number of trees)	2000
max_depth	6
scale_pos_weight	len(y_train[y_train == 0]) / len(y_train[y_train == 1])
colsample_bytree (i.e., fraction of features used by tree)	1

Table S4. XGBoost model parameters.

Parameter	Value
n_splits	5
shuffle	True
random_state	0
objective	binary:logistic
eval_metric	logloss
booster	gbtree
scoring	precision

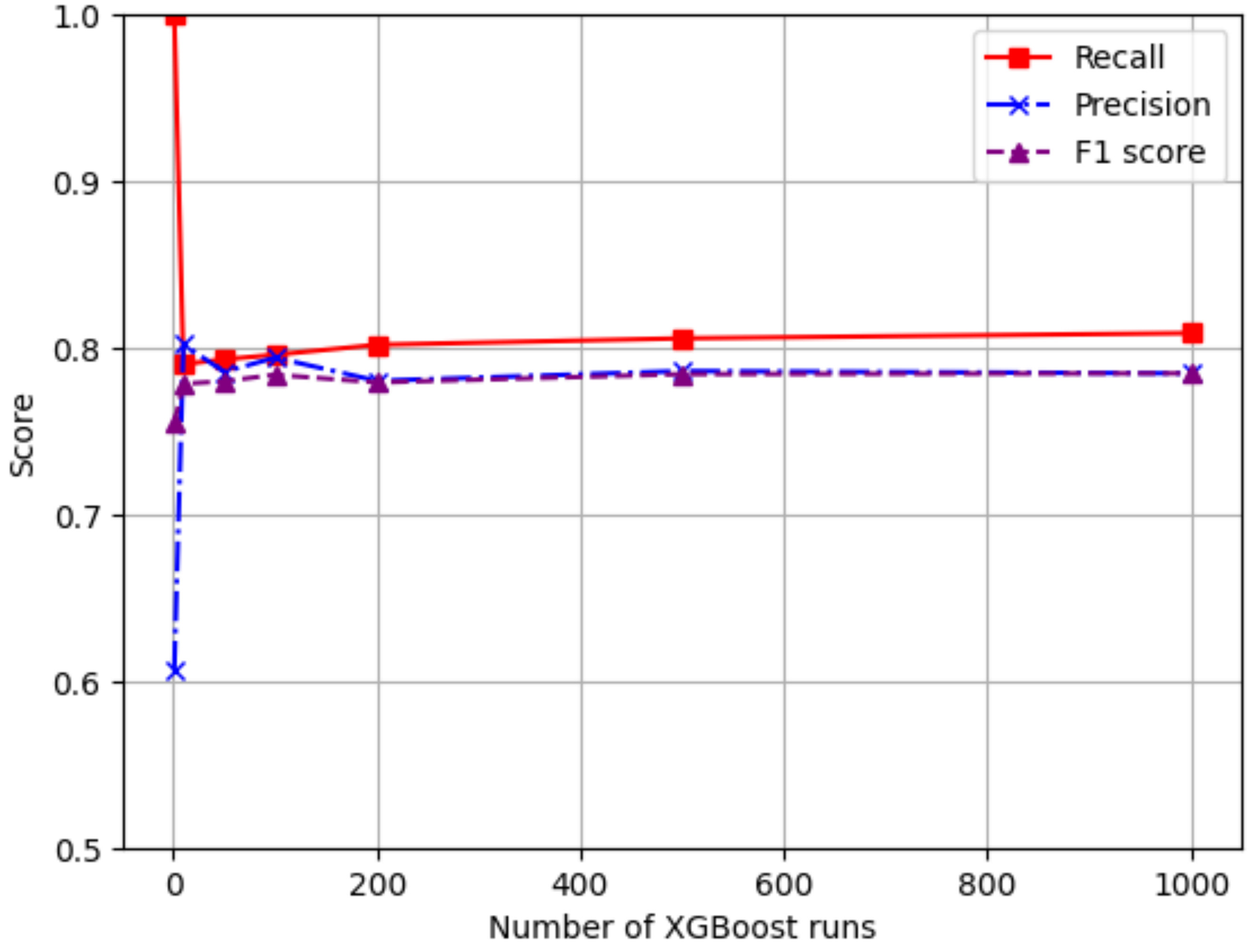

Figure S5. Number of XGBoost epochs and resultant average evaluation metric scores (F1 score, recall, and precision). Up to 500 iterations, the average scores improve slightly, but they do not improve after 500 runs, so we choose 500 runs for our final suite of models.

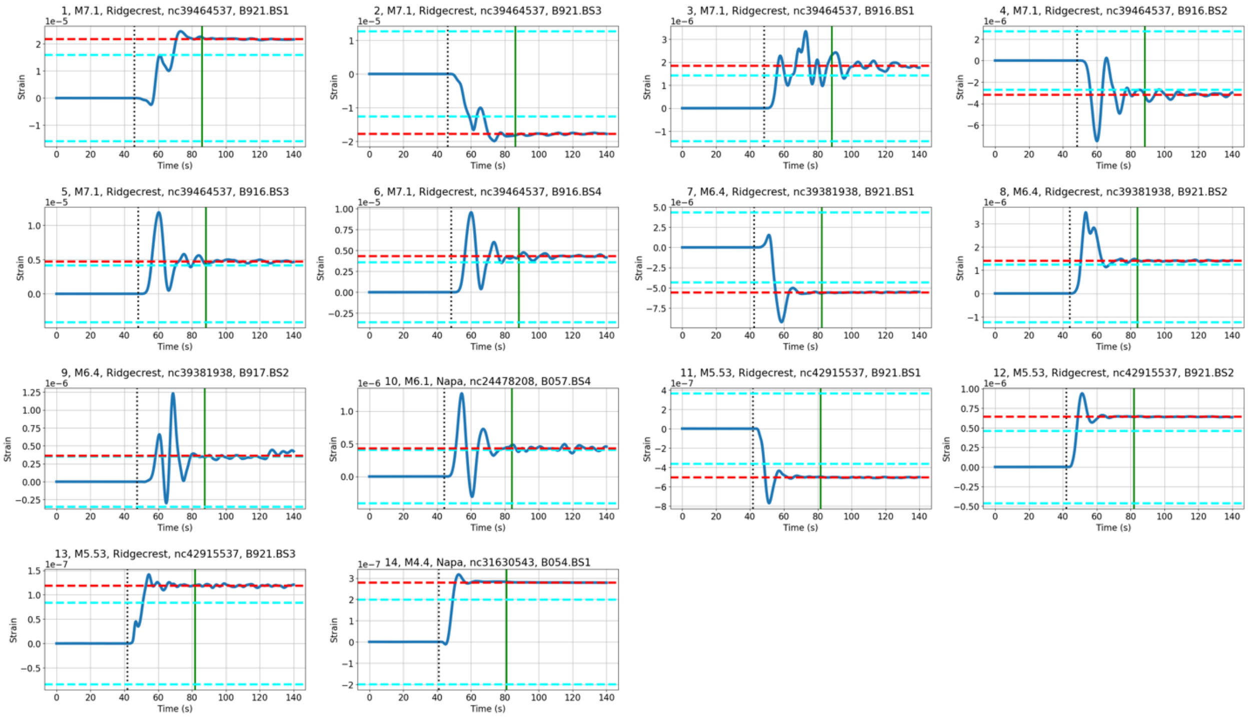

Figure S6. Borehole strainmeter waveforms from M>4.4 earthquakes with visible static offsets in the dataset we tested. A four-pole lowpass Butterworth filter at 1 Hz is applied. Although there are records with suggestions of static offsets, we define them to 'visible' if the new baseline strain is above or below 1.5 times the standard deviation of the overall strain (dashed horizontal cyan lines). The new baseline (dashed horizontal red line) is the mean strain beginning 40 seconds after the manual P-wave pick (the dotted vertical black line is at the P-wave pick, and the bold vertical green line is 40 seconds after).
