# Supplementary material for: Rapid earthquake magnitude classification via P-wave strains from borehole strainmeters and Distributed Acoustic Sensing
Source: Nat Commun. 2026 Jun 3;17:4776. doi: 10.1038/s41467-026-72223-z (PMC13233862; doi:10.1038/s41467-026-72223-z)
Supplement: Supplementary file 2 — Description of Additional Supplementary File [file 41467_2026_72223_MOESM2_ESM.pdf]

Description of Additional Supplementary Files:

Supplementary Data 1:

Naming scheme (short name and description) for all 127 features used in first iterations of XGBoost. The numbers after “Morlet” are the center frequency and the bandwidth frequency, where the center frequency indicates the primary frequency the wavelet is designed to detect, and the bandwidth frequency indicates how wide the wavelet is, with a larger number indicating a wider, less focused wavelet.
